# Compositionally-Driven Formation Mechanism of Hierarchical Morphologies in Co-Deposited Immiscible Alloy Thin Films

**DOI:** 10.3390/nano11102635

**Published:** 2021-10-08

**Authors:** Max Powers, James A. Stewart, Rémi Dingreville, Benjamin K. Derby, Amit Misra

**Affiliations:** 1 Department of Material Science and Engineering, University of Michigan, Ann Arbor, MI 48105, USA; maxpow@umich.edu; 2 Energetic Materials Dynamic & Reactive Science Department, Sandia National Laboratories, Albuquerque, NM 87185, USA; jstewa@sandia.gov; 3 Center for Integrated Nanotechnologies, Sandia National Laboratories, Albuquerque, NM 87185, USA; rdingre@sandia.gov; 4 Center for Integrated Nanotechnologies, Los Alamos National Laboratory, Los Alamos, NM 87545, USA; benderby@lanl.gov

**Keywords:** physical vapor deposition, composition modulation, phase ordering, phase-field modeling

## Abstract

Co-deposited, immiscible alloy systems form hierarchical microstructures under specific deposition conditions that accentuate the difference in constituent element mobility. The mechanism leading to the formation of these unique hierarchical morphologies during the deposition process is difficult to identify, since the characterization of these microstructures is typically carried out post-deposition. We employ phase-field modeling to study the evolution of microstructures during deposition combined with microscopy characterization of experimentally deposited thin films to reveal the origin of the formation mechanism of hierarchical morphologies in co-deposited, immiscible alloy thin films. Our results trace this back to the significant influence of a local compositional driving force that occurs near the surface of the growing thin film. We show that local variations in the concentration of the vapor phase near the surface, resulting in nuclei (i.e., a cluster of atoms) on the film’s surface with an inhomogeneous composition, can trigger the simultaneous evolution of multiple concentration modulations across multiple length scales, leading to hierarchical morphologies. We show that locally, the concentration must be above a certain threshold value in order to generate distinct hierarchical morphologies in a single domain.

## 1. Introduction

**Figure 1:**
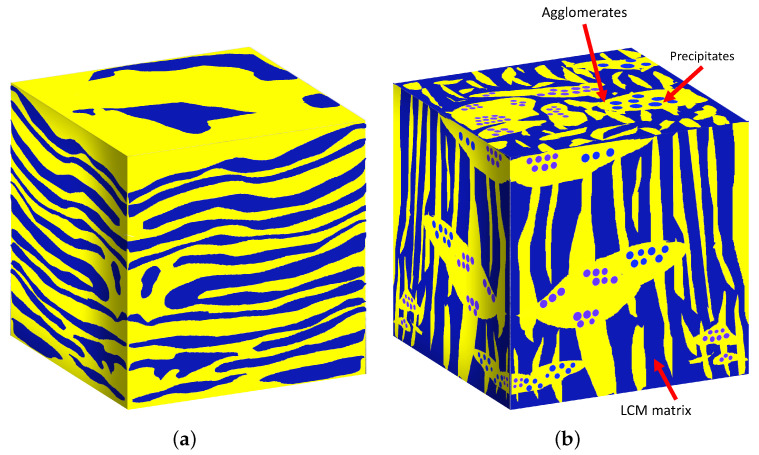
Schematic illustrations of sample microstructure morphologies that can be produced experimentally with phase separated regions (**a**) monomodal concentration modulation structure (Monomodal structure); (**b**) multimodal hierarchical microstructure.

Plasma-based, physical vapor deposition (PVD), such as sputter deposition, for instance, is a fabrication technique widely used to deposit nanostructured thin films. Depending on the elemental composition and deposition conditions, PVD is known to produce self-organized, phase-separated, nanoscale regions during the co-deposition of immiscible alloys at an elevated temperature, where the concentration modulation (CM) direction may be oriented laterally (LCM), vertically (VCM), or randomly (RCM) [1,2] with respect to film growth direction. These concentration modulations are classified as monomodal, maintaining a consistent microstructure morphology throughout the film. Several mesoscale models employed classical thin-film growth theory [3,4,5,6,7,8] to elucidate and understand the relevant kinetic and thermodynamics pathways [9,10,11,12,13], leading to the formation of such monomodal concentration modulation. Recent experimental results present a fourth class of concentration modulation with regions self-organizing into hierarchical microstructures [11,14,15]. These microstructures are multimodal, having multiple, distinct sets of features across several length scales. In Figure 1, schematics of monomodal and hierarchical structures are displayed. As shown in the schematic in panel (b), hierarchical structures in binary immiscible alloys typically display significant agglomerates with embedded nanoprecipitates, while the remainder of the thin film consists of equimolar, monomodal concentration modulations. The nanoprecipitate-rich region is termed nanoprecipitate concentration modulation (NPCM).

As discussed in previous studies [16], such hierarchical morphologies offer promising emerging functionalities, since, for instance, their unique, multi-scale phase organization enables both the suppression of shear bands and a homogeneous distribution of stress when deformed. However, the exact mechanism during deposition leading to the formation of these multimodal hierarchical morphologies is presently unknown, as *operando* observations of thin-film microstructure evolution during PVD interrupts the competitive growth mechanisms at play and hampers further microstructural evolution [17].

A key characteristic of experimental multimodal hierarchical structures is the local spatial composition heterogeneity seen near the surface of the deposited thin film. Post-deposition experimental characterization suggests that these multimodal hierarchical structures likely form via complex phase-separation thermodynamics and self-organization processes with multiple simultaneous kinetic activities occurring across several length scales [1,15]. However, simple phase-separation and film-growth mesoscale models lack the ability to capture these concurrent and multimodal morphology developments. Indeed, in most of the existing phase-field models for thin-film growth, the deposition process is simulated by adding a single bulk layer of grid points of finite thickness with randomly configured material to grow the thin film and extend the computational domain while simultaneously imposing no-flux boundary conditions [8]. This numerical scheme artificially constrains the description of the growth and associated microstructure evolution process, lacking the ability to correctly describe simultaneous kinetic activities related to the solid–vapor interfacial conditions near the surface, as well as accounting for the difference between surface and bulk diffusion of the different species being deposited. Furthermore, these models completely neglect an explicit description of the ballistic transport of the vapor phase to the growing surface, thus removing the possibility to study localized surface effects such as local variations of the vapor concentration and off-axis vapor flux.

In contrast, we recently developed a generalized PVD phase-field model [13] which addresses these limitations, and we demonstrated this model’s predictability and agreement with experimentally observed microstructures across a broad class of immiscible alloys and deposition conditions in the context of standard monomodal concentration modulations. In parallel, in our previous experimental observations [14,15], we studied a range of binary and ternary immiscible systems and identified which systems exhibited hierarchical (as opposed to the commonly observed monomodal) microstructures under elevated temperature co-deposition. Based on these previous computational and experimental studies, we are particularly interested in understanding what factors control the formation of multimodal hierarchical morphologies during deposition. To address this question, we postulate that multimodal hierarchical structures form via a multi-step, concurrent kinetic pathway in immiscible binary alloy systems that can be attributed to nuclei (i.e., small clusters of atoms) that form on the surface and that are inhomogeneous in composition. The freshly-deposited inhomogeneous nuclei form on the surface due local variability in the composition of the vapor phase composition. Such a (discrete) atomistic phenomenon can be emulated in the phase-field method by simulating localized, non-equimolar vapor regions near the surface of the growing film. As such, on the one hand, NPCM morphology evolves where, locally, the free energy dictating the phase separation process is skewed. On the other hand, in places near the film surface where the vapor phase fraction remains near equimolar, VCM/LCM nanostructures can form. Taken together, the result of this localized effect is a compositional driving force that encourages nanoprecipitate formation of a minority phase for the less-mobile elements “trapped” within agglomerates, while the remainder of the film, which remains at nearly equimolar composition, evolves into the typical monomodal concentration modulation structures.

To test this hypothesis and fully comprehend this proposed phase-separating, self-assembling hierarchical formation mechanism, we employed a joint experimental and computational approach. Experimentally, we characterized a series of Cu-Mo thin films co-sputtered at elevated deposition temperatures with the kinetic conditions noted above to encourage hierarchical organization, e.g., disparate constituent elemental mobilities and deposition at a high homologous temperature [15]. Computationally, we adapted and exercised our previously developed phase-field model to simulate PVD [13,18] to identify the factors and the necessary conditions that lead to the formation of multimodal hierarchical structures in immiscible binary alloy films and validated our predictions with the experimental characterization.

## 2. Methods

### 2.1. Phase-Field Model

We simulated the time-dependent deposition and growth of Cu-Mo thin films as a function of deposition conditions using our previously developed PVD phase-field model [13,18,19]. This phase-field model is a mesocale model that captures the dynamics of film growth and the associated evolution of concentration modulations comprising the growing film’s microstructure by describing the microstructure with a system of continuous variables that evolve both in space and time. This mesoscale model has been shown to capture a broad range of experimentally observed monomodal concentration modulations dependent on inputted deposition conditions and deposition parameters. The model explicitly accounts for major aspects of the PVD process, including: (i) the deposition of the incident vapor phase of a binary alloy onto the substrate, (ii) surface interdiffusion, and (iii) the subsequent phase ordering in the resulting inhomogeneous thin film. In this model, the total free-energy functional used to describe the microstructure–evolution dynamics is a function of three order parameters, one describing vapor transport to the thin film’s surface (ρ), one describing the vapor–solid transition and the growing thin film (ϕ), and the last one characterizing the compositional ordering and sub-surface phase separation process (*c*) during thin film growth. The time evolution of these order parameters is given by the following expressions:

(1) $$\frac{\partial \rho }{\partial t}=\nabla \cdot [D_{\rho }\nabla \rho ]-\nabla \cdot (\rho v)-\nabla \phi \cdot (\rho v)\;,$$

(2) $$\frac{\partial \phi }{\partial t}=\nabla \cdot [M_{\phi }\left(\phi \right)\nabla \frac{\delta F}{\delta \phi }]+\nabla \phi \cdot (\rho v)\;,$$

(3) $$\frac{\partial c}{\partial t}=\nabla \cdot [M_{c}(\phi ,c)\nabla \frac{\delta F}{\delta c}]\;.$$

Here, Equation (1) is the convection–diffusion equation describing vapor transport to the growing thin film’s surface along with a sink that removes any vapor that has been converted to solid. The quantity, $D_{\rho }$, is the diffusivity of the vapor, while v is the velocity field of the incident vapor (i.e., magnitude and angle, as measured from the substrate normal). These two parameters effectively account for the energy and direction of the sputtered atoms. For the evolution of the growing thin film in Equation (2), we solve the Cahn–Hilliard equation with a source term (second term in the right-end side of the equation) that couples the thin-film growth to the vapor transport, thus allowing for film growth at the expense of the incident vapor. The quantity, $M_{\phi }\left(\phi \right)$, is the Cahn–Hilliard mobility, which is taken to be constant here. The evolution of the compositional field in Equation (3) is the standard Cahn–Hilliard equation, where $M_{c}\left(\phi ,c\right)$ is a structurally and compositionally dependent Cahn-Hilliard mobility. The mobility function $M_{c}$ effectively enforces separate diffusivities on the surface and in the bulk of the thin film, $M_{i}^{\mathrm{Bulk}}$ and $M_{i}^{\mathrm{Surf}}$, which was shown to be a crucial component for the formation of different concentration modulations and surface roughness profiles at different deposition rates. This term takes the form:

(4) $$\begin{array}{c} M_{c}\left(\phi ,c\right)=M^{\mathrm{Bulk}}+M^{\mathrm{Surf}}\;, \end{array}$$

where

(5) $$\begin{array}{cc} M^{\mathrm{Bulk}} & =\frac{1}{4}(2-\phi ){\left(1+\phi \right)}^{2}[h\left(c\right)M_{A}^{\mathrm{Bulk}}+\left(1-h\left(c\right)\right)M_{B}^{\mathrm{Bulk}}]\;,\\ M^{\mathrm{Surf}} & =\exp(-{\left(\phi /\sigma^{\mathrm{Surf}}\right)}^{2})[h\left(c\right)M_{A}^{\mathrm{Surf}}+\left(1-h\left(c\right)\right)M_{B}^{\mathrm{Surf}}-M^{\mathrm{Bulk}}]\;. \end{array}$$

In Equation (4), it should be noted that h(c) and the ϕ-dependent coefficients are smooth interpolation functions between phases A and B and their respective bulk ($M_{i}^{\mathrm{Bulk}}$) or surface ($M_{i}^{\mathrm{Surf}}$) mobilities. The free-energy functional, F, in both Equations (2) and (3), is the same as in Ref. [13] without the elasticity contribution. As discussed in Ref. [13], elastic effects are second-order effects, and therefore, the coupling of the phase separation with elastic residual stresses is ignored in the present work. Further details on this model can be found in Ref. [13]. We distinguish our present phase-field model from earlier models simulating phase-separating systems, such as Ref. [8] for instance, by its ability to explicitly couple the deposition process (via ρ) with the phase-separation, and surface/bulk kinetic processes in the growing thin film (via *c* and ϕ). Combining these features with a set of inputs matching experimental deposition parameters is necessary to simulate multimodal morphologies, whereas previous studies only effectively modeled monomodal morphologies.

We numerically solved the set of coupled partial differential equations describing the deposition process and microstructure evolution in Equations (1)–(3) using the finite-difference method with second-order central difference stencils for all spatial derivatives. Since our hypothesis is that local differences in the A–B composition of the film, along with the relative disparities in the species’ mobilities, give rise to a hierarchical morphology (i.e., a concentration feature or an agglomerate feature), we performed qualitative simulations to elucidate relevant pattern formation mechanisms with this initial condition (discussed below) and did not attempt to make them quantitative with respect to a specific length and time scale. As such, all simulations were performed on a uniform, two-dimensional mesh of 512×512 grid points with dimensionless numerical and physical parameters for simplicity and efficiency, where the spatial discretization was Δx=Δy=1 and the temporal discretization was $\Delta t=10^{-2}$ for numerical stability. To determine the surface and bulk mobilities, we used surface diffusivity values for Cu and Mo based on observations from experimental depositions [20] and converted them into mobility values based on their ratio (normalized with respect to Cu) to be used in our phase-field model. The surface diffusivity values chosen for Cu and Mo were $2.5\times 10^{-1}$ m${}^{2}$·s${}^{-1}$ and $3.9\times 10^{-5}$ m${}^{2}$·s${}^{-1}$, respectively. Based on previous experimental observations [15], these choices were motivated by specific deposition conditions that accentuate the difference in constituent element mobility. Therefore, we used the dimensionless surface mobility values of 1.0 and $1.56\times 10^{-4}$ for Cu and Mo, respectively. The inputted bulk mobilities for both species were set to be an order of magnitude smaller than the corresponding surface mobility values. This means that four distinct mobilities were used, as was built and detailed into our original PVD model [13], to incorporate the separate kinetics for surface and bulk diffusion for the Cu and Mo species. Such consideration stems from Monte Carlo studies by Atzmon, Adams and coworkers [5,6,7] on atomic diffusion during film growth, who showed that surface diffusion is the primary mode of material flux as the bulk diffusion is limited to the extent that a “frozen bulk” assumption is permitted. As such, the spatial discretization was chosen to enable us to capture the characteristic sizes governed by the competition between bulk and surface ordering [7], where the bulk characteristic length scale could be defined as $\ell_{\mathrm{bulk}}=\sqrt{2D_{\mathrm{bulk}}\tau }$ and the surface characteristic length scale was defined as $\ell_{\mathrm{surf}}=\sqrt{2D_{\mathrm{surf}}\lambda /\nu }$, where $D_{i}$ are the bulk and surface diffusivities, τ is the dwell time associated with the deposited species, and λ is the surface layer thickness. Finally, we note that, by adimensionalizing the surface and bulk diffusivity with respect to the mobility of the fastest element, we could resolve the fastest diffusive time scale associated with the development of the sought-out hierarchical structures.

We simulated a dimensionless deposition rate of ν=0.8, which, based on the size of our computational domain and diffusivity parameters selected, corresponded to a deposition regime that yields a VCM thin-film microstructure, as observed experimentally. We simulated three different deposition conditions. The first condition corresponded to the deposition of a homogeneous 50-50 at.% alloy composition in the vapor for which we did not expect hierarchical structure formation. The second and third deposition conditions simulated the local formation of inhomogeneous nuclei near the surface of the growing film by introducing a local imbalance in the phase composition in the vapor near the surface of the thin film. For these two conditions, we considered that the vapor composition was at 90-10 at.% composition for a localized region of the computational domain, while for the remainder of the computational domain, the vapor was at a 50-50 at.% composition. We considered a small and large localized region with this configuration for the imbalance in the vapor composition in these second and third deposition conditions, respectively. By assuming sub-domains with distinct vapor compositions within the computational domain, we created a variation of the free-energy landscapes near the surface of the growing film. Within the equimolar region, the film microstructure could therefore self-assemble as LCM/VCM, etc., depending on the deposition parameters; however, in regions (or sub-domains) where the vapor composition was non-equimolar near the surface of the thin film, the free-energy landscape was skewed and became locally penalized, leading to the formation of NPCM microstructures in the growing film.

### 2.2. Synthesis and Characterization of Co-Sputtered Cu-Mo Thin Films

To complement and provide credence to our simulated predictions, we compared our simulation results to experimental PVD co-sputtered Cu-Mo thin films. We used Cu (99.999%) and Mo (99.95%) 2” targets to co-deposit Cu-Mo thin films, with the Cu set at 250 W and the Mo target set at 110 W, onto an oxidized Si substrate rotating at 20 rpm. We applied a 50 W RF bias with a 5” throw distance from the targets to deposit three distinct films at differing temperatures: 600, 700, and 800 ${}^{\circ }$C. The base pressure at room temperature of $6.5\times 10^{-8}$ Torr increased slightly after heating the substrate to 600 ${}^{\circ }$C and was measured to be $1.7\times 10^{-7}$ Torr at 600 ${}^{\circ }$C using a Kurt J. Lesker 392 Series Wide-Range Combination gauge. The targets were oriented above the substrates in a “sputter-down”, off-axis configuration. The off-axis angle was approximately 30 degrees. The sample was biased before and during deposition to pre-clean the substrate and enhance film density and adhesion, respectively. Power was provided to each target with individual direct-current power supplies and power was constantly maintained throughout the deposition process. We used this configuration to deposit 1 μm-thick films at a combined Cu-Mo deposition rate of 0.8 nm·s${}^{-1}$. This deposition rate has been shown to yield the formation of VCM microstructures [2,11]. We characterized cross-sectional and planar foils of the films using a JEOL 3100R05 double-Cs corrected scanning transmission electron microscope (STEM) (Japan Electron Optics Laboratory Company, Limited, Tokyo, Japan), operated at 300 keV with a point-to-point resolution of 0.055 nm and a convergent angle of 111${}^{\circ }$ for high angle darkfield (HAADF) imaging and an 8 cm camera length for true Z-contrast imaging. Cross-sectional transmission electron microscopy foils were prepared using conventional focused ion beam preparation and placed onto a silicon TEM grid. To eliminate any influence of the FIB sectioning on the film morphology, two low-energy “cleaning,” passes with the Ga beam operated at 5 keV were made on the foil. Energy dispersive X-ray spectroscopy (EDS) compositional maps were collected using a JEOL SDD X-ray detector (Japan Electron Optics Laboratory Company, Limited, Tokyo, Japan) with a 60 mm${}^{2}$ active area.

## 3. Results and Discussion

**Figure 2:**
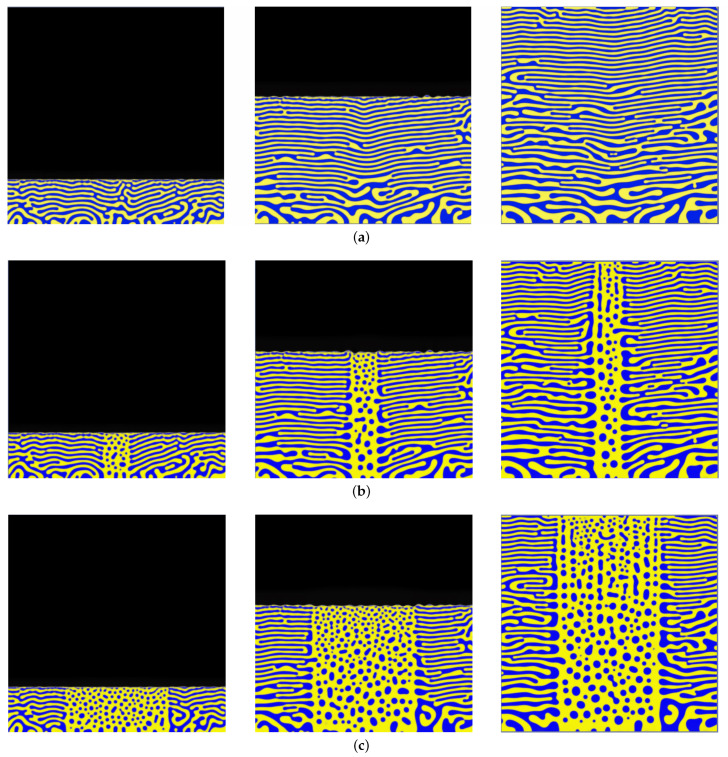
Simulated time-dependent co-deposition of two species under three compositional domains: (**a**) 50-50 at.% (ν=0.8, equimolar); (**b**) designated region in middle of mesh with 90-10 at.% and remaining area 50-50 at.% (ν=0.8, equimolar with thin region of localized vapor phase concentration); (**c**) ν=0.8, equimolar with wide region of localized vapor phase concentration, similar to (**b**) but with wider designated region. The different panel from left to right shows snapshots at different times of the simulation for given deposition conditions.

We first examine the sufficient (local) conditions for film compositional distribution leading to multimodal microstructures. In Figure 2a–c, simulated microstructures for three compositional conditions are shown for the binary system with disparate constituent mobilities at a relative deposition rate, ν = 0.8. Figure 2a represents the configuration where the vapor concentration is homogeneous at a 50-50 at.% vapor phase fraction when the co-deposited alloy lands on the substrate and initially phase separates into a locally RCM microstructure [19] (see left panel of Figure 2a). As the deposition progresses, newly-deposited material phases separate and diffuse a limited distance via surface diffusion before being buried by subsequent layers. After coarsening via bulk diffusion, the thin film yields concentration–modulation layers in the direction of film growth, i.e., VCM (see middle and right panels of Figure 2a), aligning with our previous predictions for equimolar deposition [13,21] and experimental depositions in the case of Cu-Mo thin films [11]. In contrast, as seen in Figure 2b, the presence of a local concentrated 90-10 at.% vapor region (i.e., the presence of nuclei with an inhomogeneous composition), over a small domain near the surface of the thin film drastically affects the subsurface microstructural evolution. The minority phase in the concentrated region becomes spherical and interspersed in a random spatial distribution in the majority phase. The spherical precipitates coarsen during deposition to form an NPCM structure, while the adjacent 50-50 at.% regions simultaneously form VCM. This self-organization is due to energetic minimization in both the concentrated region and the VCM structures. Within the concentrated region, the minority deposited specie with reduced mobility is unable to diffuse to the adjacent VCM regions, becoming encapsulated into spherical precipitates to reduce surface energy within the majority phase agglomerate similar to what has been noted in co-deposited Cu-Ta systems [14]. The VCM regions follow the same pathway as described for Figure 2a. For a wider local concentration region, a similar observation is noted (see Figure 2c). Again, the agglomerates/precipitates and VCM morphologies concurrently evolve during deposition. The juxtaposition of the two microstructure morphologies, NPCM and VCM, in Figure 2b,c correlates with the experimental observations of the formation of hierarchical structures, which show adjacent differing microstructure morphologies at separate length scales [15]. All microstructures in Figure 2 exhibit coarser features towards the bottom of the film via limited bulk diffusion during the solid-phase deposition.

**Figure 3:**
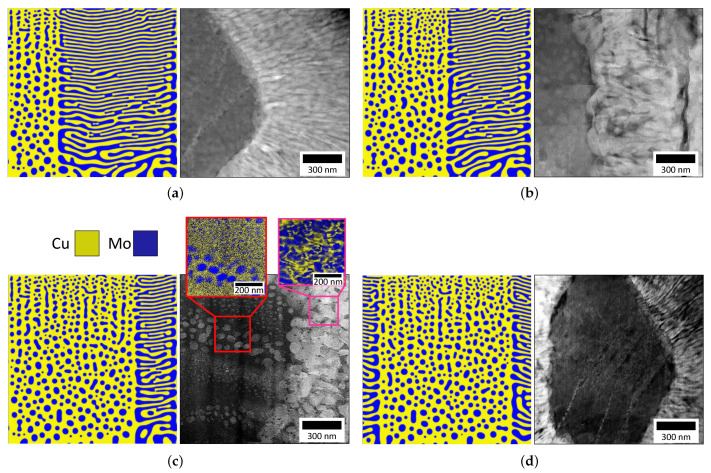
(**a**–**d**) Comparisons of STEM-HAADF images of four distinct Cu-Mo films co-deposited at 600 ${}^{\circ }$C at a flux of 0.8 nm·s${}^{-1}$ with their respective simulated microstructures with compositional domains from the experimental data. Each experimental micrograph is a magnified image interface of a Cu-rich agglomerate, including Mo nanoprecipitates, and the surrounding Cu-Mo concentration modulations. Darker contrast in HAADF shows Cu-rich regions; lighter contrast shows Mo-rich regions. EDS images of the agglomerate region and the adjoining concentration modulations showing the spatial elemental distribution in each are displayed in the inset (**c**). The corresponding simulation results were taken after the thin films were deposited to a thickness of 512 grid cells, corresponding a 1 μm-thick film.

The difference in elemental kinetics and a local imbalance in the free energy both lead to preferential agglomeration of one specie during deposition, generating a spatially inhomogeneous composition affecting microstructural evolution [14,15]. A similar compositional influence on film growth has been noted in other systems such as Zr-W thin films [22], which present similar disparity in diffusivities. To assess the concentration–morphology dependence, we now compare the microstructure of Cu-Mo films co-sputtered at 600 ${}^{\circ }$C with a deposition rate of 0.8 nm·s${}^{-1}$ with our phase-field predictions using inputs that corresponds to these experimental deposition parameters. In our model, we inserted localized, highly-concentrated vapor regions into the computational domain to mimic inhomogeous nuclei forming on the surface of the thin film during deposition and simulate the effects of preferential agglomeration. The size of these highly-concentrated vapor regions was chosen to capture similar domains observed experimentally. Each simulated microstructure depicts a differing elemental distribution, but all four simulated domains replicate the hierarchical features observed experimentally. Figure 3a–d present the cross-sectional and planar HAADF micrographs with their matching simulated microstructures for four distinct Cu-Mo films. Although the deposition parameters are the same for the four films, the micrographs emphasize different regions of the hierarchical morphology (a concentration modulation feature or an agglomerate feature). The difference in each region is driven by the localized compositional variance of the film’s Cu and Mo species, with the localized equimolar regions presenting a concentration modulation feature, while a localized disparate concentration with composition akin to 90-10 at.% presents an agglomerate. Further details on the microscopic characterization of the Cu-Mo films can be found in previous studies [11,15].

In Figure 3a, the cross-sectional HAADF micrograph reveals a darker-contrast Cu-rich region populated by lighter-contrast Mo-nanoprecipitates surrounded by alternating layers of Cu-Mo VCM. The related simulated microstructure predicts a co-deposition with a third of the domain occupied by a Cu-rich domain (90 at.% Cu-10 at.% Mo) and the remainder having an equimolar Cu-Mo concentration. The simulated microstructure evolved both regions simultaneously, coarsening the features with limited bulk diffusivity and ultimately forming the familiar hierarchical morphology with juxtaposed NPCM and VCM morphologies. In Figure 3b,c similar results are shown for similar deposition conditions. The difference is that a larger concentrated agglomerate region in the simulated microstructure with highly concentrated localized regions representing 50% and 75% of the domain are shown in Figure 3b and Figure 3c, respectively. The first EDS image inset in Figure 3c shows a size dichotomy in the Mo nanoprecipitates, with one population approximately 20 nm in diameter and another population approximately 7 nm in diameter. To a degree, while no specific size-dependent formation mechanism is implemented in our model [23], the simulated microstructures capture the binary size distribution of the nanoprecipitates evidenced in Figure 3a–d. Figure 3d and the corresponding Cu-Mo HAADF image reveal a concentrated region surrounded by two equimolar regions in VCM orientation. Our mesoscale predictions again captured the hierarchical morphology, suggesting the boundary regions have a negligible effect on the evolution of the 90-10 at.% region. One noticeable difference is that in isolated cases, the experiments’ results reveal that the interface between the agglomerate and VCM region is curved, a phenomenon not observed in our simulations. Shadowing effects and a greater local variability in the composition of the vapor phase in the deposition chamber during the experimental synthesis of the thin film are factors not currently considered in our model. In our model, we inputted the experimental kinetic parameters and note a qualitative agreement between the hierarchical morphologies observed experimentally and those predicted by our simulations. In our simulation, we obtained a planar interface between the regions, since the spatial variation in the vapor phase is also planar. We note, however, that to naturally capture the actual experimental variations in the vapor distribution, our phase-field model would need to be improved to explicitly account for the separate sputtering targets present in a PVD chamber and also explicitly model the transport of sputtered atoms through the gas phase and potential re-sputtering at the surface of the film. This could be achieved, for instance, by simulating the transport of atoms from the source to the substrate using Monte Carlo simulations to predict the spatial composition of the vapor phase when combining different sources [24,25]. In this work, we simplified the problem and used a spatially varying distribution of the vapor phase as input (as a continuous field variable) to repesent inhomogeneous nuclei and show the effects of those variations on the resulting microstructure, vis a vis hierarchical microstructures. Other factors, such as the energy (and momentum) of the sputtered atoms and reflected argon neutrals, are known to affect the film growth stress and the morphology [3,26,27], e.g., inter-columnar porosity can be closed with increasing energetic bombardment and growth residual stress can transition from tensile to compressive, for ambient temperature (or low homologous temperature) deposition. In this investigation, the depositions were carried out at elevated temperatures (∼600 ${}^{\circ }$C or higher) and the observed phase-separation was presumably driven by diffusive mass transport in the deposited film. Nevertheless, there may be second-order effects due to the energetic bombardment, particularly at lower deposition temperatures. Such improvements warrant further studies and the development of our PVD model. Finally, the observed experimental microstructure is representative of the process parameters, specifically the deposition time and temperature. If the films were to be reheated after deposition and annealed at an elevated temperature for a long time, the microstructure length scale and morphology may coarsen/evolve further. The simulation could be run to study annealing of as-deposited microstructure, but experiments/simulations on the thermal stability of the hierarchical microstructure are outside the scope of this manuscript.

**Figure 4:**
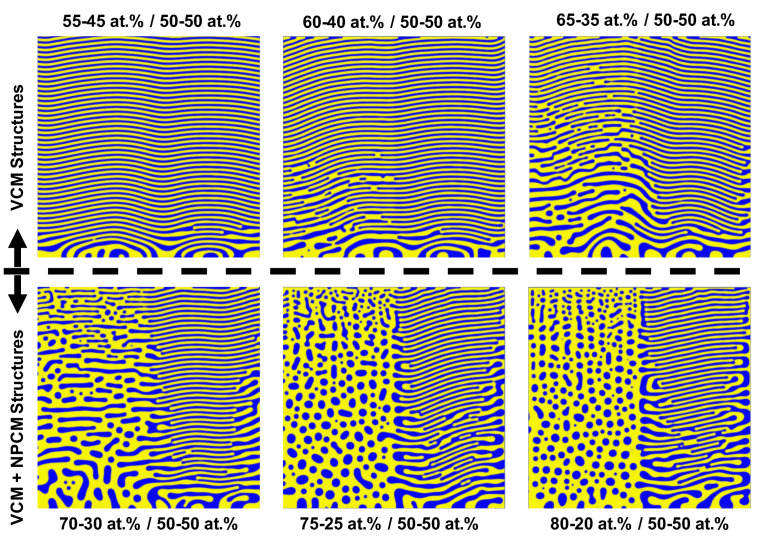
Simulated microstructures with split compositional domain with respect to x-axis. The first half of the computational domain had varied phase fraction incrementing by 5 at.%; the second half had equimolar phase fraction. Dashed line delineates the transition from a monomodal VCM to multimodal VCM + NPCM morphologies.

Finally, we examine the necessary (local) conditions for the film’s compositional distribution leading to multimodal microstructures to complement the above qualitative reasoning. We conducted a parameter sweep in terms of the concentration of localized vapor regions to determine the threshold local (non-equimolar) concentration necessary for the formation of multimodal hierarchical structures. In Figure 4, we present six simulated domains with differing vapor phase fractions in the first half of the computational domain, incrementing the vapor concentration by 5 at.% for subsequent simulations, while the second half of the computational domain remains at an equimolar concentration. The simulated phase fractions illustrated in Figure 4 show that the transition from a monomodal VCM to a multimodal, hierarchical VCM+NPCM occurs for a local vapor phase fraction between 70 and 30 at.% and 75-25 at.%. Thus, these results point to the fact that the necessary condition for multimodal hierarchical formation will only occur when the kinetics of the constituent elements generate agglomeration regions of at least 70-25 at.% phase fraction in the vapor (i.e., inhomogenous nuclei with such a phase fraction). Therefore, the experimental deposition parameters during thin-film deposition must favor the accumulation of one species during film growth to create the 70-30 at.% regions. Such preferential agglomeration may be influenced by spatial inhomogeneities of the deposited atoms in the deposition chamber as they land from the vapor phase. Kinetically, the diffusivity of the co-deposited elements can be tuned using the deposition temperature and elemental selection [28]. The conditions of low deposition rate and reduced noble gas partial pressure must be present for one species to have an advantageous surface interdiffusion length, leading to its clustering on the film surface during phase separation.

## 4. Conclusions

This work was focused on testing the hypothesis that the evolution of different morphologies at different length scales in a growing film can be explained in terms of the compositional heterogeneities near the surface of the film that are created in the early stages of the deposition due to local agglomerations that are rich in the faster diffusing species. Our results can be summarized as follows. Immiscible alloys are likely to form hierarchical morphologies if locally inhomogeneous nuclei form near the surface of the deposited film due to compositional variations within the vapor phase. We showed that their composition needs to be greater than 70-25 at.% (approximately) to locally trigger the development of such hierarchical structure. We assessed our hypothesis by exercising a generalized, mesoscale PVD model that can effectively predict the evolution of different morphologies at different length scales during vapor deposition. We simplified the problem of representing the formation of inhomogeneous nuclei by using a spatially varying distribution of the vapor phase as input (as a continuous field variable) and showed the effects of those variations on the resulting formation of potentially hierarchical microstructures. Our model incorporated the deposition behavior (vapor to solid transition) explicitly and it distinguished a bulk and surface diffusivity for each element and induces phase-separation for the species. The vapor composition was an input to the model and did not account for complex sputtering depositions mechanisms. What our model demonstrated is that local imbalance in the vapor composition near the surface of the growing film for a binary alloy with significant differences in diffusivity between the two species can lead to the formation of graded, hierarchical microstructures, while other existing models do not encompass multimodal microstructure formation. By replicating experimental conditions with this assumption, the majority of the material flux was facilitated via surface diffusion, which, combined with thin film growth kinetics, produced the multiple kinetic pathways responsible for multimodal microstructure morphologies. A logical expansion of this work would be the incorporation of more complex thermodynamic free-energy potentials and sputtering mechanisms (i.e., shadowing effects, the development of residual stresses in the film) during depositions into our phase-field model. Our experimental characterization revealed non-equilibrium phases present in multimodal hierarchical structures, indicating multiple localized minima in the free-energy functional that describes the solid phase equilibria of the system. A metastable state in the NPCM could be stabilized with a third energetic well, akin to prior simulations of three-phase C:Ni thin films [29].

## Data Availability

The data presented in this study are available on request from the corresponding author.

## Abbreviations

The following abbreviations are used in this manuscript:

PVD	physical vapor deposition
CM	concentration modulation
LCM	lateral concentration modulation
VCM	vertical concentration modulation
NPCM	nanoprecipitate concentration modulation
RCM	random concentration modulation
EDS	energy dispersive X-ray spectroscopy
HAADF	high angle darkfield
STEM	scanning transmission electron microscope

## References

[1] Heo T., Shih D., Chen L.Q. (2014). Kinetic pathways of phase transformations in two-phase Ti alloys. Metall. Mater. Trans. A.

[2] Derby B., Cui Y., Baldwin J., Misra A. (2018). Effects of substrate temperature and deposition rate on the phase separated morphology of co-sputtered, Cu–Mo thin films. Thin Solid Films.

[3] Thornton J. (1974). Influence of apparatus geometry and deposition conditions on the structure and topography of thick sputtered coatings. J. Vac. Sci. Technol..

[4] Thompson C. (1990). Stress and grain growth in thin films. Annu. Rev. Mater. Sci..

[5] Adams C., Atzmon M., Cheng Y.T., Srolovitz D. (1992). Phase separation during co-deposition of Al–Ge thin films. J. Mater. Res..

[6] Atzmon M., Kessler D., Srolovitz D. (1992). Phase separation during film growth. J. Appl. Phys..

[7] Adams C., Srolovitz D., Atzmon M. (1993). Monte Carlo simulation of phase separation during thin-film codeposition. J. Appl. Phys..

[8] Ankit K., Derby B., Raghavan R., Misra A., Demkowicz M. (2019). 3-D phase-field simulations of self-organized composite morphologies in physical vapor deposited phase-separating binary alloys. J. Appl. Phys..

[9] Galdikas A. (2008). Study of nanoclusters growth at initial stages of ultrathin film deposition by kinetic modeling. Appl. Surf. Sci..

[10] Lu Y., Wang C., Gao Y., Shi R., Liu X., Wang Y. (2012). Microstructure map for self-organized phase separation during film deposition. Phys. Rev. Lett..

[11] Derby B., Cui Y., Baldwin J., Arróyave R., Demkowicz M., Misra A. (2019). Processing of novel pseudomorphic Cu–Mo hierarchies in thin films. Mater. Res. Lett..

[12] Kairaitis G., Galdikas A. (2020). Modelling of phase structure and surface morphology evolution during compound thin film deposition. Coatings.

[13] Stewart J., Dingreville R. (2020). Microstructure morphology and concentration modulation of nanocomposite thin-films during simulated physical vapor deposition. Acta Mater..

[14] Powers M., Derby B., Shaw A., Raeker E., Misra A. (2020). Microstructural characterization of phase-separated co-deposited Cu–Ta immiscible alloy thin films. J. Mater. Res..

[15] Powers M., Derby B., Manjunath S., Misra A. (2020). Hierarchical morphologies in co-sputter deposited thin films. Phys. Rev. Mater..

[16] Cui Y., Derby B., Li N., Mara N., Misra A. (2018). Suppression of shear banding in high-strength Cu/Mo nanocomposites with hierarchical bicontinuous intertwined structures. Mater. Res. Lett..

[17] Xie T., Fu L., Qin W., Zhu J., Yang W., Li D., Zhou L. (2018). Self-assembled metal nano-multilayered film prepared by co-sputtering method. Appl. Surf. Sci..

[18] Stewart J., Spearot D. (2016). Phase-field models for simulating physical vapor deposition and grain evolution of isotropic single-phase polycrystalline thin films. Comput. Mater. Sci..

[19] Herman E., Stewart J., Dingreville R. (2020). A data-driven surrogate model to rapidly predict microstructure morphology during physical vapor deposition. Appl. Math. Model..

[20] Oura K., Katayama M., Zotov A., Lifshits V., Saranin A. (2003). Elementary Processes at Surfaces II. Surface Diffusion. Surface Science: An Introduction.

[21] Krzanowski J. (2004). Phase formation and phase separation in multiphase thin film hard coatings. Surf. Coat. Technol..

[22] Borroto A., García-Wong A., Bruyère S., Migot S., Pilloud D., Pierson J., Mücklich F., Horwat D. (2021). Composition-driven transition from amorphous to crystalline films enables bottom-up design of functional surfaces. Appl. Surf. Sci..

[23] Johnson W. (2001). Spinodal decomposition in a small radially stressed sphere. Acta Mater..

[24] Van Aeken K., Mahieu S., Depla D. (2008). The metal flux from a rotating cylindrical magnetron: A Monte Carlo simulation. J. Phys. D Appl. Phys..

[25] Depla D., Leroy W. (2012). Magnetron sputter deposition as visualized by Monte Carlo modeling. Thin Solid Films.

[26] Thornton J., Hoffman D. (1989). Stress-related effects in thin films. Thin Solid Films.

[27] Petrov I., Barna P., Hultman L., Greene J. (2003). Microstructural evolution during film growth. J. Vac. Sci. Technol..

[28] Langer G., Erdélyi G., Erdélyi Z., Csiszár G. (2019). Determination of diffusion coefficients in immiscible systems: CuW as an example. Materialia.

[29] Kairaitis G., Galdikas A. (2020). Mechanisms and dynamics of layered structure formation during co-deposition of binary compound thin films. Coatings.

